# Crowdsourced Perceptions of Human Behavior to Improve Computational Forecasts of US National Incident Cases of COVID-19: Survey Study

**DOI:** 10.2196/39336

**Published:** 2022-12-30

**Authors:** David Braun, Daniel Ingram, David Ingram, Bilal Khan, Jessecae Marsh, Thomas McAndrew

**Affiliations:** 1 Department of Psychology Lehigh University Bethlehem, PA United States; 2 Actuarial Risk Management Austin, TX United States; 3 Computer Science and Engineering Lehigh University Bethlehem, PA United States; 4 College of Health Lehigh University Bethlehem, PA United States

**Keywords:** crowdsourcing, COVID-19, forecasting, human judgment

## Abstract

**Background:**

Past research has shown that various signals associated with human behavior (eg, social media engagement) can benefit computational forecasts of COVID-19. One behavior that has been shown to reduce the spread of infectious agents is compliance with nonpharmaceutical interventions (NPIs). However, the extent to which the public adheres to NPIs is difficult to measure and consequently difficult to incorporate into computational forecasts of infectious diseases. Soliciting judgments from many individuals (ie, crowdsourcing) can lead to surprisingly accurate estimates of both current and future targets of interest. Therefore, asking a crowd to estimate community-level compliance with NPIs may prove to be an accurate and predictive signal of an infectious disease such as COVID-19.

**Objective:**

We aimed to show that crowdsourced perceptions of compliance with NPIs can be a fast and reliable signal that can predict the spread of an infectious agent. We showed this by measuring the correlation between crowdsourced perceptions of NPIs and US incident cases of COVID-19 1-4 weeks ahead, and evaluating whether incorporating crowdsourced perceptions improves the predictive performance of a computational forecast of incident cases.

**Methods:**

For 36 weeks from September 2020 to April 2021, we asked 2 crowds 21 questions about their perceptions of community adherence to NPIs and public health guidelines, and collected 10,120 responses. Self-reported state residency was compared to estimates from the US census to determine the representativeness of the crowds. Crowdsourced NPI signals were mapped to 21 mean perceived adherence (MEPA) signals and analyzed descriptively to investigate features, such as how MEPA signals changed over time and whether MEPA time series could be clustered into groups based on response patterns. We investigated whether MEPA signals were associated with incident cases of COVID-19 1-4 weeks ahead by (1) estimating correlations between MEPA and incident cases, and (2) including MEPA into computational forecasts.

**Results:**

The crowds were mostly geographically representative of the US population with slight overrepresentation in the Northeast. MEPA signals tended to converge toward moderate levels of compliance throughout the survey period, and an unsupervised analysis revealed signals clustered into 4 groups roughly based on the type of question being asked. Several MEPA signals linearly correlated with incident cases of COVID-19 1-4 weeks ahead at the US national level. Including questions related to social distancing, testing, and limiting large gatherings increased out-of-sample predictive performance for probabilistic forecasts of incident cases of COVID-19 1-3 weeks ahead when compared to a model that was trained on only past incident cases.

**Conclusions:**

Crowdsourced perceptions of nonpharmaceutical adherence may be an important signal to improve forecasts of the trajectory of an infectious agent and increase public health situational awareness.

## Introduction

Forecasting the transmission of infectious agents can support decisions made by public health officials and key decision makers [1,2]. Past forecasts of seasonal influenza, Ebola, dengue, chikungunya, and Zika have helped officials take short-term action to stymie the spread and burden of disease and draft policy decisions [3-8]. The COVID-19 pandemic has further highlighted the importance that forecasts play in support of public health situational awareness [9-11].

The majority of forecasts of an infectious disease are generated by computational models; however, past work has shown that human judgment is also capable of making accurate predictions of a diverse number of phenomena [12,13], including infectious agents [14-17].

Work in human judgment predictions can be categorized into direct and indirect predictions. Direct predictions are collected by asking humans to estimate the probability of a future event of interest. Researchers have used various methods to solicit direct predictions from a lay, expert, or mixed crowd by varying the format humans use to submit predictions and training different algorithms to combine individual forecasts [18-21]. Structured elicitation formalizes how a prediction should be collected to minimize potential biases or undue influences, and a researcher could use several different protocols to rigorously collect predictions [19,20,22].

Past work has found middling performance when asking those with subject matter expertise to make direct predictions [23,24]. As with experts, the performance of predictions made by lay people has been mixed, and the variability in predictive performance is likely due to cues in the environment that are related to the event of interest [25], as well as people’s reliance on heuristics to make fast decisions with little information [26-29]. Humans are subject to several cognitive biases that negatively impact our ability to make sound judgments [30,31]. That said, there are many examples where predictions based on mental heuristics outperformed computational models [32].

Work on aggregating direct human judgment predictions has focused on adjusting for correlated predictions between individuals, assessing the number of individual predictions to combine, and determining how to appropriately weight individuals based on past predictive performance [18,21]. Direct predictions take advantage of a human’s ability to build a prediction from available structured data and information typically unavailable to a computational model, such as subjective information, intuition, and expertise [33].

Indirect predictions of a future event are collected by (1) extracting human judgment data from a passive source such as social media [34-37], (2) actively asking a crowd about covariates that may be related to the target of interest, or (3) asking a crowd to take actions in a prediction market, which can be mapped to probabilistic predictions [38,39]. Indirect predictions offer an opportunity to train a statistical model on both measured objective data and subjective data.

Past work that incorporated social media data in a model often mapped behaviors to a set of random variables and included these random variables in a statistical model [34-37,40]. Most studies have framed these human and social media sources as passive signals that can be mined to contribute to more accurate forecasts. For example, a recent study leveraged mobility data gathered from Twitter to improve forecasts of incident COVID-19 cases at multiple geographic levels [41]. Digital interaction and engagement data beyond social media may be useful predictive signals as well, as a recent study found that Google search trends related to COVID-19 symptoms improved both nowcasting and forecasting of COVID-19 incident cases and deaths [42]. Compartmental models have also been proposed that take into account human behavior by estimating the contact network between individuals, and the reproductive and recovery rates, or by building a more complicated function between disease states that takes into account human behavior [43]. Prediction markets are another approach for aggregating human judgment, which ask a pool of participants to place bets on the potential of future events with an incentive for each participant to optimize their total earnings [38,39]. The goal of creating a prediction market is not to link behavior to outcomes of interest but to take advantage of an individual’s ability to extract alternative data sources that are not accessible to computational models and respond to the aggregate behaviors of a market. Models that include indirect predictions report improved performance compared to models that do not include indirect predictions; however, performance varies by the infectious agent and type of data collected. Human behavior and perceptions can also be used to predict social media engagement and community behavior that might benefit decision-making of policy makers and community leaders. For example, past work has looked at which types of messages from organizations shared on Twitter foster the strongest public engagement [44], as well as which sources for health-related information are likely to be sought out based on demographics and how these factors contribute to adherence to social distancing guidelines [45].

In this work, we study how crowdsourced questions related to nonpharmaceutical interventions (NPIs) in one’s community can contribute to an improved forecast of COVID-19 incident cases at the national level. We posed 21 questions related to NPIs to a representative sample from the United States over a period of 36 weeks. These crowdsourced data were used to estimate the association between perceptions of adherence to NPIs and incident cases at the US national level 1-4 weeks in advance. In addition, we fit a predictive model and showed that adding crowdsourced data on perceptions of adherence improves forecast accuracy for incident cases when compared to a control that does not include perceptive data.

To the current literature, we contribute a novel data stream of community-scale perceptive information [46] that shows (1) strong associations with incident cases 1-4 weeks ahead at the national level and (2) improved predictive accuracy of out-of-sample predictions 1-3 weeks ahead when included in a computational model.

## Methods

### Ethical Considerations

We obtained retroactive clearance from Lehigh University’s institutional review board (IRB) to publish the data (#1808500-1). The IRB determined obtaining informed consent was not necessary because the data were recorded in such a manner that the identity of human subjects cannot be readily ascertained directly or through identifiers linked to the subjects. Data that have been made publicly available are similarly deidentified [46]. Participants completed surveys either (1) on a volunteer basis or (2) in exchange for compensation. Compensated participants earned credits from the survey platform that could be redeemed for gift cards or donated to charity.

### Survey Logistics

#### Participants and Recruitment

There were 10*,*852 responses to the survey over the course of 36 weeks starting August 30, 2020, and ending April 28, 2021 (281 responses per week on average with an SD of 119). Paid participants were initially recruited through the *SurveyMonkey* platform (4405*/*10*,*852, 40*.*5%) from September 23, 2020, through February 15, 2021. SurveyMonkey is a survey platform with access to more than 140 million participants globally. The platform requires a fee per service and comes with assurance that paid participants will be a representative sample from the locale of interest. A survey can be sent to a set of participants who meet specific criteria (called a targeted audience), such as country of origin, age, socioeconomic factors (income, marital status, and employment), etc. Participants in this study were required to reside in the United States and be at least 18 years old. Survey design, distribution, and data collection were managed via SurveyMonkey software.

From February 16, 2021, to April 27, 2021, participants were recruited from the *Pollfish* survey platform (3295*/*10*,*852, 30*.*4%). This change was made due to SurveyMonkey delivering a highly variable number of responses per week and, in some weeks, failing to deliver the number of responses ordered. Pollfish is another fee per response survey platform that allows the researcher to specify a targeted audience and guarantees a representative number of responses. The goals and services of SurveyMonkey and Pollfish are similar, though Pollfish software collects higher resolution spatial data about respondents. The Pollfish platform collected responses from participants who met the same criteria as those for SurveyMonkey.

Compensated respondents from SurveyMonkey and Pollfish accounted for approximately 70% (7700/10,852, 71.0%) of the responses, and the final approximately 30% (3152*/*10*,*852, 29.0%) of participants were recruited as volunteers and participated through the SurveyMonkey platform from August 30, 2020, to April 28, 2021. These volunteers were mostly recruited via word of mouth and social media.

We removed participant responses from the analysis if (1) more than half of the questions (ie, 11 of the 21 questions) were left blank or had a response of “Don’t know” (4*.*7% [511*/*10*,*852] of responses) or (2) a participant gave the same response to every question (2*.*3% [331*/*10*,*852] of responses). All blank and “Don’t know” responses were excluded from the analysis (9*.*7% [20*,*569*/*214*,*200] of total question responses [ie, *N_Participants_*×21]).

#### Survey Timeline

A total of 36 weekly surveys were sent to participants beginning on September 6, 2020, and ending on April 30, 2021. Surveys were distributed to unique participants each Monday, Wednesday, and Friday, and surveys were closed on Sundays. Surveys were not sent to the same participant more than once in a week.

SurveyMonkey surveys were open to participants for compensation from the 4th week of the survey period (September 2020) to the 21st consecutive week of the survey (February 2021), and SurveyMonkey surveys were open to volunteers over the entire 36-week survey period. Pollfish surveys were open to participants from the 21st week of the survey period (February 2021) until the 36th consecutive week of data collection (the end of the survey period; April 2021).

In July and August 2020, surveys were sent to participants to (1) fill out the survey and (2) solicit feedback about whether the questions asked in the survey were worded clearly. Feedback from these first 2 pilot surveys was used to update and finalize surveys sent between September 2020 and April 2021.

#### Survey Content and Questions

Surveys between September 2020 and April 2021 asked participants to answer the same set of 21 “core” questions (see Textbox 1 for a list of core questions). Core questions asked participants about their perceptions of their community members’ adherence to NPIs, such as mask wearing, and their adherence to public health guidelines related to testing, quarantine, and large gatherings. Participants gave responses to survey questions on a Likert scale with the following options: “None/not adopted,” “Few/20%,” “Some/40%,” “Many/80%,” “All/100%,” and “Don’t know.”

In addition to the 21 core questions, several weeks included topical questions asking participants about their perceptions of behavior during specific events (eg, the size of holiday gatherings). Because these questions were not consistent throughout the duration of the study, we chose not to include them in the analyses. At the end of the survey, participants were also asked for optional thoughts and feedback about how COVID-19 is being addressed in their community and how the survey may be improved in the future (for summary reports of the data composed in real time, see a previous report [47]).

The order in which questions were presented was randomized across all 21 questions in the Pollfish surveys, and SurveyMonkey questions were randomized within 5 categories that asked participants about individual NPI behaviors, adherence to guidelines associated with community businesses, testing and quarantine, awareness, and restrictions or policies related to educational institutions (see Multimedia Appendix 1).

**List of the 21 “core” questions that were presented to participants in every survey from September 6, 2020, to April 30, 2021.**

**Questions**

What percent of people in your community do you notice are usually:

1. Wearing a mask in public

2. Maintaining social distance

3. Staying at home

How common is it in your community for:

4. Restaurants to have reduced seating

5. Businesses to be closed – work from home only

6. Hairdressers and barbers to be open with restrictions

7. Visitors to senior living facilities to be restricted

8. Commonly touched surfaces to be sanitized

9. Hospitals to have special protection in areas that treat COVID patients

In your community, how common is it for people to follow recommendations or requirements to:

10. Get tested for active virus

11. Get antibody testing to detect prior infection

12. Quarantine people who have been in close contact with people with positive tests

13. Quarantine people with positive tests

14. Quarantine travelers from higher infection places

15. Limit large gatherings of people

How many people in your community are aware of:

16. Local level of COVID infections

17. Statewide targets for reducing COVID spread

18. Local approach to limiting COVID spread

In your state, what percent of:

19. Colleges are closed or holding only remote classes

20. Schools (K-12) are closed or holding only remote classes

21. Violations of COVID restrictions result in fines or police enforcement

#### Data Acquisition and Availability

Survey data were acquired retrospectively from a team of actuaries (Daniel Ingram and David Ingram) who were interested in the study of human behavior, crowdsourcing, and how perceptions may be predictive of the spread of SARS-CoV-2. There were several limitations to survey collection: (1) participant identifiers were not collected longitudinally and so we cannot track individuals who contributed to the survey, and (2) the wording of survey instructions was slightly different across the SurveyMonkey and Pollfish platforms, which could bias responses.

Individual respondent data of all 21 questions for all 36 weeks are available in a previous report [46]. The data are in wide format where each row represents a single survey response, and columns are present for the date the survey was completed and the 21 answers to survey questions.

We obtained approval from Lehigh University’s IRB to publish these data on an open-source platform.

### Epidemiological Data

Incident cases per epidemiological week (epidemic week) at the national level were collected from the Johns Hopkins University CSSE GitHub repository [48]. This repository stores cumulative cases per day from January 22, 2020, to the present for all 50 states and a set of 5 territories. To compute incident cases for day *D*, we subtracted cumulative cases at day *D* from cumulative cases at day *D*+1. We computed incident cases for day *D* at the national level by summing incident cases for all 50 states and all 5 territories. Daily incident cases at the national level were summed to arrive at incident cases per epidemic week, where an epidemic week began on Sunday and ended on Saturday.

### Assessing Whether the Crowd was Representative of the US Population

We assessed graphically whether our sample was representative of the US population by plotting for all states (*s*) the pair (*r_s_,e_s_*), where *r_s_* is the total number of observed participants for state *s* and *e_s_* is the estimated expected number of responses from state *s*.

Our estimate *e_s_* assumes that *r_s_* was drawn from a random variable *R_s_* ∼ Bin(*N,θ_s_*), where *N* is the total number of participants across all surveys and *θ_s_* is the probability of choosing at random a citizen registered in state *s*. We estimated *θ_s_*, 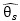, as the census estimate for state *s* divided by the sum of census estimates for all states. The value *e_s_* is 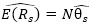.

We included an estimated correlation coefficient between the observed and expected number of participants sampled across all states. For each state, we also compared the relative difference between the observed and expected proportions of participants (Multimedia Appendix 2).

### Statistical Setup

We suppose a survey response to question *q* from participant *i*, at time *t*, *x_t,i,q_*, was generated from a random variable *X_t,i,q_* which has support *supp*(*X_t,i,q_*) = {0*,*1*,*2*,*3*,*4} corresponding to 5 different levels of adherence. The value 0 corresponds to no adherence or adherence not adopted in the community, and the value 4 corresponds to complete adherence (the response “All/100%” on the survey). Random variables at time *t* for question *q* between 2 participants are considered independent.

Mean perceived adherence (MEPA) is defined for a specific question *q* and at a specific time *t* as the average of *x_t,i,q_* over participants, or



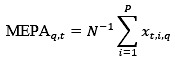



where *N* is the number of responses for question *q* at time *t*. MEPA*_q,t_* is intended to measure an aggregated adherence to a specific type of NPI. Though individual responses are discrete, MEPA*_q,t_* is a continuous value. If we define the random variable MEPA*_q,t_* as the average of *N* independent random variables with finite variance, then we expect MEPA*_q,t_* to have a bell-curved distribution that resembles the normal distribution restricted to the closed interval from 0 to 4.

Incident US national COVID-19 cases at epidemiological week *t*, (*c_t_*), are assumed to be generated from a corresponding random variable *C_t_*, and we make no additional assumptions about this time series.

### Estimating the Correlation Between MEPA and Incident Cases

For each survey question, we estimated the correlation coefficient between MEPA at epidemiological week *t* and US national incident cases at epidemiological week *t*, *t*+1, *t*+2, *t*+3, and *t*+4. Line lists of the estimated correlation coefficient at each week-ahead time point and 95% CIs are available in Multimedia Appendix 3.

### Clustering Questions

We fit a hierarchical clustering algorithm to all 21 MEPA time series for 2 through 10 clusters. Dissimilarity between 2 time series was computed using the Euclidean distance. The Silhouette coefficient was used to assess the quality of fitting 2 clusters, 3 clusters, and so on (up to 10 clusters) [49]. A dendrogram was plotted to visualize the clustering, and MEPA time series were grouped and plotted over the epidemiological week.

### Forecast Models With and Without Crowdsourced Perceptions

#### SIR Plus Vector Autoregression Moving Average

An SIR (susceptible, infected, and removed) model was fit to the number of US incident cases to produce an estimated number of incident cases *I_t_*, and residuals (*ϵ_t_*=*c_t_*−*I_t_*) were modeled with a vector autoregression moving average (VARMA) model that included one or more MEPA time series.

The SIR model estimates at time *t* the number of individuals existing in the susceptible (*S_t_*), infected (*I_t_*), and removed (*R_t_*) compartment according to



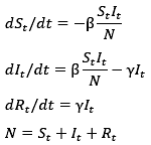



with initial values *S*_0_*, I*_0_*,* and *R*_0_, and parameters *β>*0 and *γ>*0. We chose *S*_0_ equal to the number of individuals in the United States, according to the most recent census. The initial value *I*_0_ was set equal to the reported number of infections for the first epidemiological week in which survey data were collected (August 30, 2020, to September 05, 2020), and *R*_0_ was set to 0. The initial value problem above was integrated by the Runge-Kutta-Fehlberg method, and parameters *β* and *γ* were estimated by minimizing the least squares solution between *I_t_* and the reported number of incident cases (estimates of the SIR model at 4 different time points can be found in Multimedia Appendix 4).

Residuals were generated as *e_t_*=*c_t_*−*I_t_*, and we assumed that these residuals together with one of the MEPA time series can be modeled as a VARMA model. VARMA assumes the residuals, and the MEPA time series *M_q_* follows

*θ*(*L*)*Y_t_ = ψ*(*L*)*U_t_*

where *Y_t_*=[*ϵ_t_*, *m_q,t_*]*'*, *U_t_* is a random vector following a white noise process or *U_t_* ∼ N(0*,*Σ), the operator *θ*(*L*)=*B*_1_*L*+*B*_2_*L*^2^+··· and *B_k_* is a matrix of coefficients, the operator *ψ*(*L*)=*A*_1_*L*+*A*_2_*L*^2^ +··· and *A_k_* is a matrix of coefficients, and the operator *L^j^* is the lag operator or *L^j^Y_t_*=*Y_t_*_−_*_j_*. We assumed the covariance between any *Y_s_* and *Y_t_* is fixed and equal to Σ.

The optimal number of lags for *θ* and for *ψ* was estimated every week through each of the 36 weeks by computing the Akaike information criterion (AIC) for models fit with all combinations of 1 through 3 lags for *θ* and 1 through 3 lags for *ψ*. The combination that resulted in the lowest AIC was picked.

#### SIR Plus Random Forest Plus VARMA

To incorporate all MEPA time series into a model, we first fit an SIR model to the original time series and computed the residuals *e_t_*=*c_t_*−*I_t_*. Next, we trained a random forest regression *f* with 5000 trees, where the desired output is *ϵ_t_* as a function of *e_t_*_−1_, and all the MEPA time series values, smoothed using LOWESS, with a lag of 1. The residuals *δ_t_*=*e5;_t_*−*f*(*e_t_*_−1_,*M*^ˆ^_1_*_,t_*_−1_*,M*^ˆ^_2_*_,t_*_−1_*,*··· *,M*^ˆ^_21_*_,t_*_−1_), where *M*^ˆ^*_q,t_* is the LOWESS smoothed MEPA time series value for question *q* at time *t*, were computed and were assumed to follow an autoregressive integrated moving average (ARIMA) process, or *θ*(*L*)*δ_t_*=*ψ*(*L*)*u_t_*. Lags were chosen at each week based on the AIC in the same manner as with the above SIR plus VARMA model.

#### Control Model

Our control model followed the same SIR “detrending” of the original incident case time series and then fit an ARIMA to the residuals. The ARIMA followed a similar approach as the VARMA model when modeling

*Y_t_* ∼ *e_t_*

*Θ*(*L*)*Y_t_=ψ*(*L*)*u_t_*

where *u_t_* ∼ *N*(0, σ^2^). The only addition to this model is that we may “difference” *Y_t_* by successively subtracting the values of *Y* at time *t-1* from the values of *Y* at time *t* for all times. The difference computes *d_t_*=∇*Y_t_*=*Y_t_* −*Y_t_*_−1_, fits the model above, generates forecasts of *d_t_*_+1_*, d_t_*_+2_*,*···, and then recovers *Yt*+*l* by computing *Y*(*t*+*l*)−1 + *d*(*t*+*l*).

The ARIMA process is a first attempt model in many time series applications. If models that include MEPA variables cannot improve upon the above SIR plus ARIMA model, then MEPA may not add any predictive value over using lagged values of incident cases alone.

The above VARMA and ARIMA models were fit using the *statsmodels* package in Python [50].

### Predictive Scoring

Forecasts were scored using the weighted interval score (WIS) over *K* central quantiles [51].



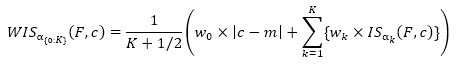



where the interval score (IS*_αk_*) is



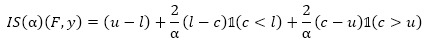



and where *F* is a predictive cumulative distribution function, 1(*x*) is an indicator function, the value *u* represents the (1–*α/*2) quantile of *F*, *l* represents the *α/*2 quantile of *F*, *m* represents the median or 0.50 quantile, and *c* is the eventually reported truth [52]. Moreover, weight *w*_0_ equals 1/2 and *w_k_*=α_k_/2.

The WIS and interval score are negatively sensed, with larger values indicating worse predictive performance compared to smaller values. The best possible WIS is 0, and the worst possible WIS is positive infinity.

## Results

### Overview

Comparison of the response rates across the 2 survey platforms (ie, SurveyMonkey and Pollfish) revealed that sample sizes each week were consistently higher following the switch to Pollfish. The sample was mostly geographically representative of the US population with slight oversampling in the Northeast. MEPA values were more variable at the beginning of the survey period than at the end, suggesting either that responses became more consistent over time or that larger sample sizes throughout the survey period resulted in lower response variability. A clustering analysis revealed that survey questions could be clustered into 4 groups based on question type, suggesting that future surveys might be more efficient by targeting these question types using fewer questions. A correlation analysis revealed reasonably strong correlations between several MEPA time series and incident COVID-19 cases 1-4 weeks ahead. Several MEPA time series also increased the predictive accuracy of a forecasting model of incident COVID-19 cases 1-4 weeks ahead.

### Survey Platform Response Rates

**Figure 1:**
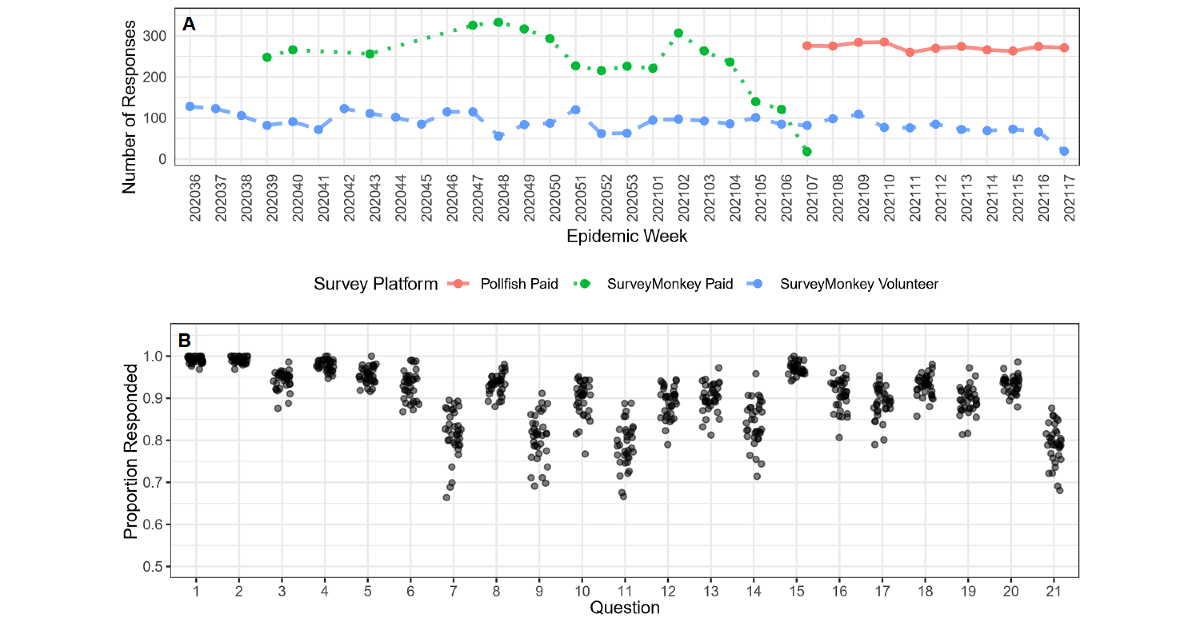
(A) The number of participant responses per epidemic week for the Pollfish platform (red) and for those who submitted responses on SurveyMonkey who were compensated (green) and who were volunteers (blue). (B) The proportion of participants who responded to each question in a given epidemic week. Volunteers made consistent contributions each week as did the Pollfish participants who were compensated, while the number of compensated participant contributions on the SurveyMonkey platform varied. Questions with a lower proportion of responses corresponded to those questions that asked about nonpharmaceutical intervention behaviors that were more difficult to observe, such as visitation rules at senior living facilities (question 7), whether members of the community received antibody testing (question 11), and quarantine of recent travelers (question 14).

SurveyMonkey surveys received an average of 236*.*06 (SD 81*.*14) compensated responses per week and an average of 88*.*80 (SD 22*.*68) volunteer responses per week, revealing that response rates for paid surveys were higher but more variable across weeks than volunteer survey responses. Pollfish surveys received an average of 272*.*55 (SD 7*.*80) compensated responses per week, and volunteer responses were not collected on the Pollfish system. Overall, sample sizes each week were consistently higher following the switch to Pollfish (Figure 1A).

### Question Response Rates

The mean percentage of questions that a participant answered was 87*.*89% (SD 6*.*15%) (Figure 1B). Questions 1 through 5 and question 15 were answered on average 94*.*98% (SD 1*.*47%) of the time, while questions 7, 9, 11, 14, and 21 had the lowest probability of responses, with an average response rate of 78*.*63% (SD 2*.*09%).

### Representative Sampling

States from which most responses were collected included California (956/10,120, 9.5%), New York (876/10,120, 8.7%), Pennsylvania (678/10,120, 6.7%), Texas (645/10,120, 6.4%), and Florida (456/10,120, 4.5%).

The correlation between the observed frequency of responses and expected frequency was 0*.*90 (95% CI 0*.*84-0*.*94; *P<.*001) and suggested that the response rates were proportional to the population at the state level. We compared for each state the proportion of observed responses to the proportion of individuals in that state according to the census (see Multimedia Appendix 2 for the observed proportion, expected proportion, and relative difference).

**Figure 2:**
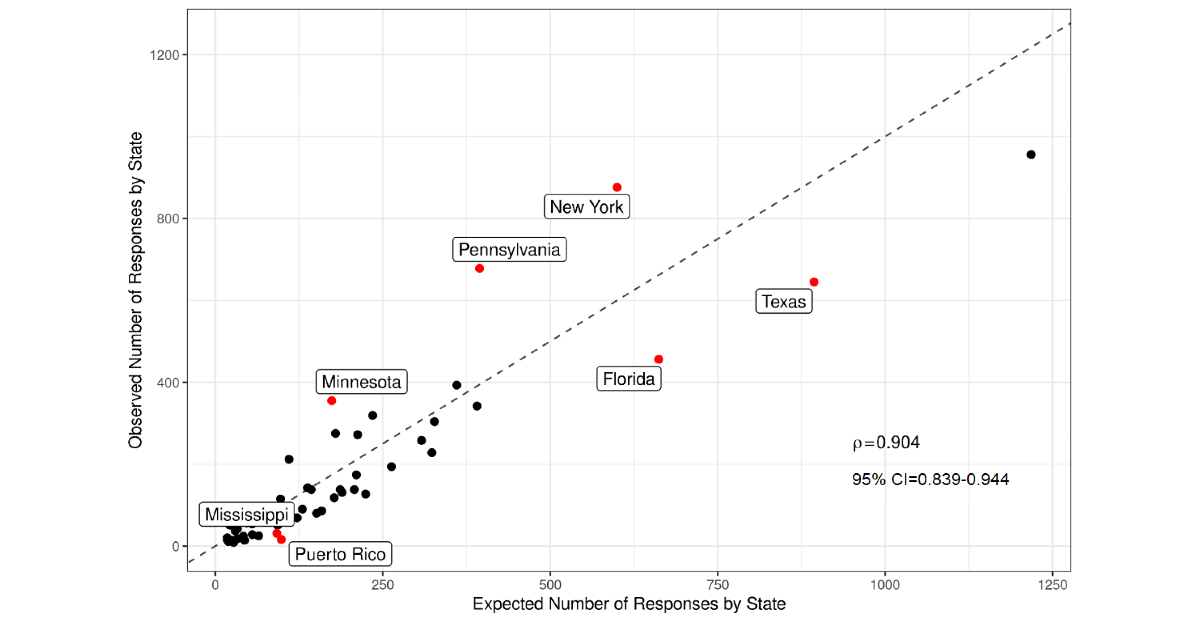
The number of observed responses to the surveys summed over the survey period (vertical axis) compared to the expected number of total responses according to the census (horizontal axis). The dashed line indicates if the observed and expected numbers of responses equal one another. Some states are oversampled and undersampled.

Seven states deviated from the expected response rates by more than 9 SDs. Four states were underrepresented (Mississippi, Puerto Rico, Florida, and Texas), and 3 states were overrepresented (Minnesota, Pennsylvania, and New York) (Figure 2). Pennsylvania was the most overrepresented state.

When both compensated and volunteer responses were included, the response frequency in Pennsylvania was 10 SDs above the expectation and when volunteer responses were removed the response frequency decreased to 3.5 SDs below the expectation.

To assess how switching survey platforms in the midst of data collection may have impacted the results, we analyzed whether the representativeness of the sample changed depending on the survey platform. We computed the average relative difference between expected and observed responses across all states, and compared this measure across survey platforms. This analysis revealed that the state residency of paid participants (ie, not volunteers) from SurveyMonkey was more representative of the US population (mean −0.599, SE 0.015) compared with the state residency of paid participants from Pollfish (mean −0.751, SE 0.019; *t*_51_=7.58; *P*<.001).

### MEPA Over Time

**Figure 3:**
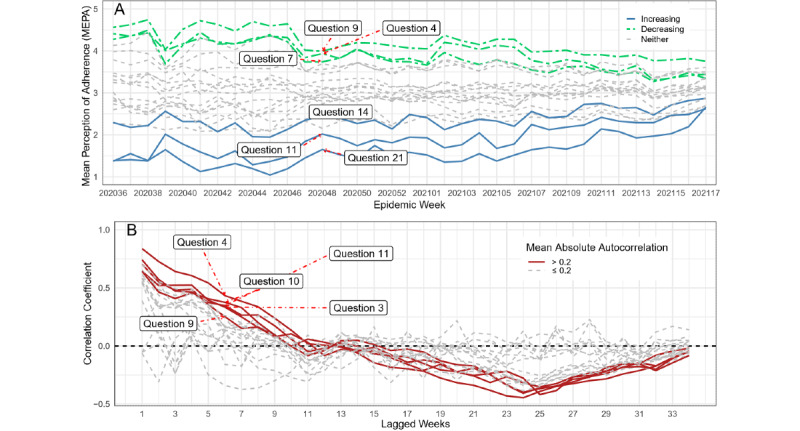
(A) Mean perception of adherence (MEPA) for 21 questions asked over the survey period. (B) Autocorrelation for all 21 MEPA time series for a lag of 1 to 34 weeks. Perceptions of adherence for questions that asked about state policies (question 21) and antibody testing practices (question 11) show an increase over the survey period, while perceptions of adherence for questions that asked about restrictions placed on senior living facilities (question 7) and restaurants (question 4) show a decrease. Mean absolute autocorrelations for 5 questions across 34 lagged weeks are above 0.2. The estimated correlation between MEPA values at time t and t−l is greater than 0.35 for lags of up to 4 weeks (l=4) for a majority of MEPA time series. MEPA time series appear to contain more structure than a random walk, suggesting that crowdsourced perceptions may be a useful signal for predicting incident cases.

MEPA increased the most from the start to the end of the survey for the following 3 questions: question 21 (∆mean_21_=mean_21,week36_ – mean_21,week1_=1.29) that asked participants about knowledge of their state policies and whether “violations of COVID restrictions result in fines or police enforcement;” question 11 (∆mean_11_=1*.*24) that asked how frequently community members follow recommendations to seek “[...] antibody testing to detect prior infection;” and question 14 (∆mean_14_=0*.*57) that asked participants how frequently members of their community quarantine after traveling (Figure 3A).

MEPA decreased the most from the start to the end of the survey for the following 3 questions: question 7 (∆mean_7_=−1.07) that asked participants how frequently restrictions are placed on visiting senior living facilities; question 4 (∆mean_4_=−0.83) that asked how frequently restaurants have reduced seating capacity; and question 9 (∆mean_9_=−0.81) that asked about the frequency of special protection in hospitals when treating patients with COVID-19.

The SD between MEPA values at the beginning of the survey period (SD_beginning_=0*.*89) was larger than the SD between MEPA values at the end of the survey period (SD_end_=0*.*33) (Figure 3A). The mean MEPA value over all 21 questions remained similar over the course of the survey (mean_beginning_=3*.*15, mean_end_=3*.*14). This result could be due to either a convergence in perceptions over time or reduced variability due to increased sample sizes throughout the survey period.

The estimated correlation between MEPA values at time *t* and *t*−*l* was greater than 0.35 for lags of up to 4 weeks (*l*=4) for a majority of MEPA time series (Figure 3B) and suggested that many MEPA time series contain more structure than a random walk. Responses to the following 5 survey questions had a mean absolute autocorrelation greater than 0.2: question 3 ([...] staying at home), question 4 ([...] restaurants complying with Centers for Disease Control and Prevention [CDC] recommendations to have reduced seating), question 9 ([...] special protection in hospital areas that treat COVID patients), question 10 ([...] get tested for active virus), and question 11 ([...] get antibody testing to detect prior infection). The mean absolute autocorrelation for these 5 questions across 34 lagged weeks was above 0.2. A more detailed view of autocorrelation for a lag of 1 week has been provided in Multimedia Appendix 5.

### Clustering Questions According to Similarities in Responses Over Time

**Figure 4:**
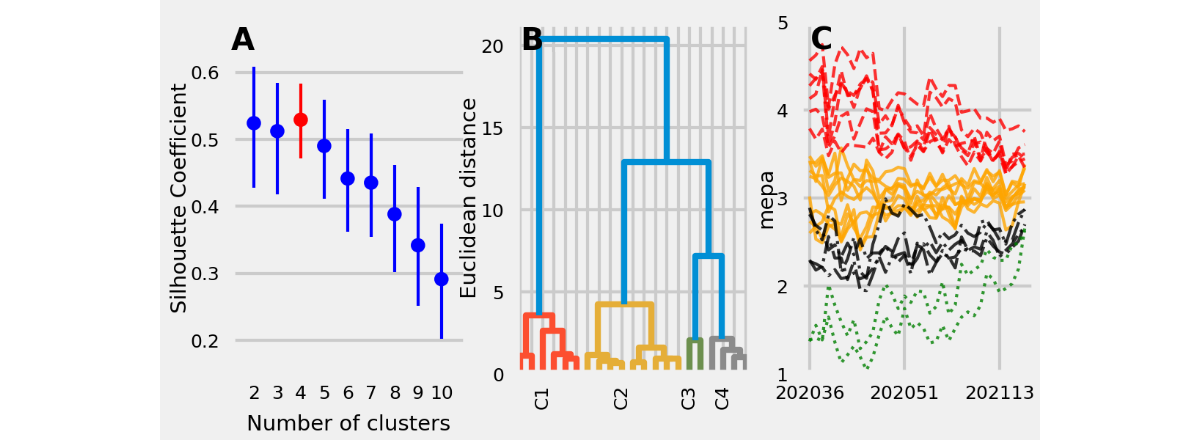
Hierarchical clustering of 21 mean perception of adherence (MEPA) time series using Euclidean distance as a measure of dissimilarity between 2 time series. (A) Silhouette coefficients for 2-10 clusters of MEPA time series. (B) Dendrogram that reports questions on the horizontal axis and dissimilarity between individual questions or clusters on the vertical axis. (C) MEPA time series clustered into 4 groups corresponding to the highest silhouette coefficient. Because MEPA time series can be separated into similar groups, a smaller survey may be able to capture the same patterns of the US public’s perceptions of adherence to nonpharmaceutical interventions.

MEPA time series were grouped into the following 4 clusters (Figure 4A and B): (1) cluster of questions with values between 2.5 and 3.5 (ie, low to medium adherence; Figure 4C), (2) cluster with values that decreased over time (Figure 4C), (3) cluster with values near 2.25 at the beginning of the survey and that increased over time (Figure 4C), and (4) cluster with values near 1.25 at the beginning of the survey and that increased over time, ending above 2.50 by the end of the survey (Figure 4C).

Cluster quality as measured by the silhouette coefficient was the highest when grouping MEPA time series into 4 clusters; however, the silhouette coefficient for 4 clusters was similar to the silhouette coefficient for 2 and 3 clusters (Figure 4A). In the cluster in Figure 4C, there may exist 2 clusters—one with increasing adherence over time and another with decreasing adherence over time.

MEPA time series within the same cluster asked participants about similar adherence behaviors. Questions corresponding to avoidance behaviors (questions 2, 12, and 15) were more similar to one another than the other questions, as were questions that asked about limitations to businesses (questions 4 and 6), awareness of the high infectivity rate of the virus at a local level (questions 2 and 13), and awareness at the state level (questions 16 and 17). These results suggested that participants might have considered groups of questions in similar ways (eg, those related to avoidance), which suggests that future surveys might benefit from targeting these factors more directly.

For autocorrelations between MEPA responses 1-4 weeks ahead across the different clusters, see Multimedia Appendix 6.

### Correlation Between Perceptions of Adherence and Reported Incident Cases

**Figure 5:**
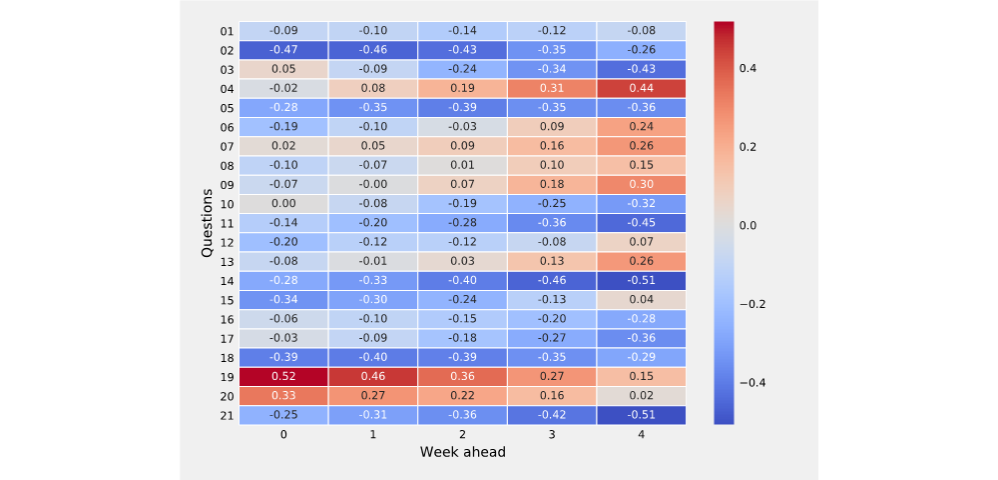
Linear correlation between 21 mean perception of adherence (MEPA) time series associated with questions about the perception of adherence and incident cases 1-4 weeks ahead at the US national level. The correlation between question 2 that asked “What percent of people in your community do you notice are usually wearing a mask in public?” and incident cases 1-4 weeks ahead was −0.26 or lower, and the correlation between question 19 that asked “In your state, what percent of colleges are closed or holding only remote classes?” and cases 1-3 weeks ahead was 0.27 or higher. Select crowdsourced perceptions of adherence to nonpharmaceutical interventions correlated with short-range and long-range reported incident cases at the national level.

The estimated correlation (*ρ*) between the MEPA time series representing responses to the question “What percent of people in your community do you notice are usually maintaining social distance?” and incident cases 1 week ahead was −0*.*46 (95% CI −0*.*69 to −0*.*15). Moreover, the correlation (*ρ*) was −0*.*3 (95% CI −0*.*67 to −0*.*12) for incident cases 2 weeks ahead, −0*.*35 (95% CI −0*.*61 to −0*.*02) for those 3 weeks ahead, and −0*.*26 (95% CI −0*.*55 to 0*.*08) for those 4 weeks ahead (Figure 5). The MEPA time series for the question “In your state, what percent of colleges are closed or holding only remote classes?” had an estimated correlation (*ρ*) of 0*.*46 (95% CI 0*.*15 to 0*.*69) for cases 1 week ahead. Moreover, the correlations (*ρ*) were 0*.*36 (95% CI 0*.*04 to 0*.*62), 0*.*27 (95% CI −0*.*07 to 0*.*55), and 0*.*15 (95% CI −0*.*19 to 0*.*46) for reported incident cases 2 weeks, 3 weeks, and 4 weeks ahead, respectively, at the US national level (Figure 5, row 19). Correlation coefficients and 95% CIs for each question are available in Multimedia Appendix 3. Taken together, these results show that changes in the perceptions of NPI compliance (ie, MEPA time series) are associated with changes in COVID-19 incident cases.

### Out-of-Sample Improvement in Forecasting With the Crowdsourced MEPA

**Figure 6:**
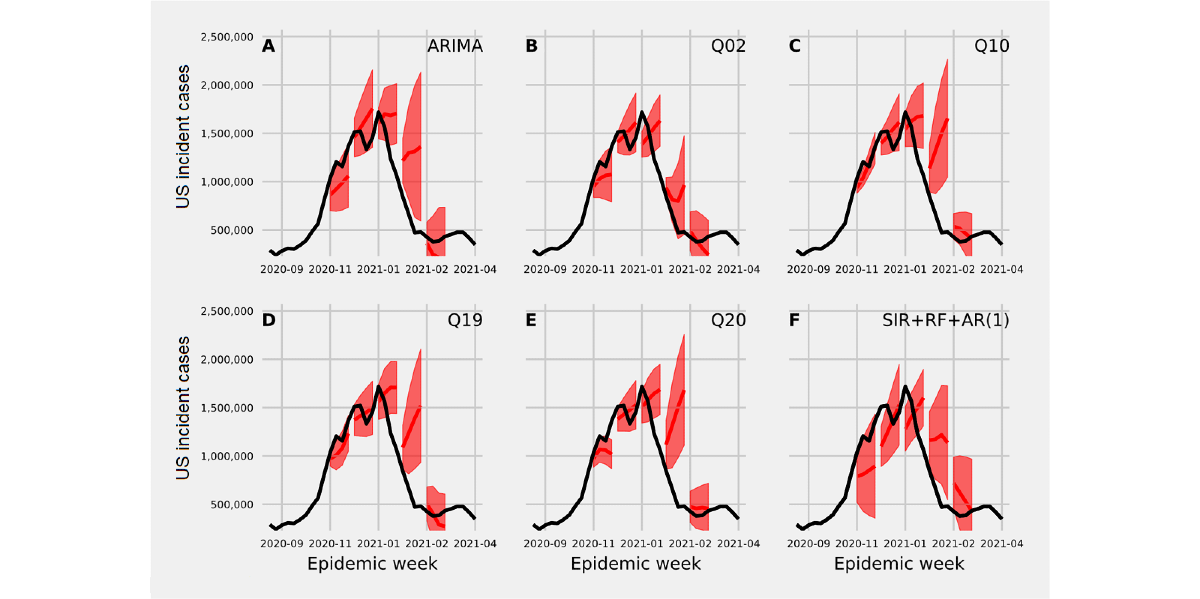
Forecasts of US national incident cases 1-4 week ahead at 6 time points throughout the survey period by first fitting an SIR (susceptible, infected, and removed) model and then modeling the residuals by (A) fitting an autoregressive model with 1 lag, (B-E) fitting a vector autoregression moving average that includes the residual time series and mean perception of adherence (MEPA) values for select questions, and (F) fitting a random forecast to residuals including MEPA values for all questions asked of participants plus an AR(1) model. AR(1): autoregression with lag of 1; ARIMA: autoregressive integrated moving average; RF: random forest.

Models that included both historical counts of US national incident cases and MEPA data changed the forecast trajectory and the width of prediction intervals compared to a model that only took into account the past time series of incident US national cases (Figure 6). The model that included historical counts and a random forecast regression incorporating all MEPA data proposed a similar trajectory to the ARIMA (control) model that included only case data, had wider prediction intervals before the peak of reported cases, and had a smaller prediction interval just after the peak of reported cases (Figure 6A and F).

**Figure 7:**
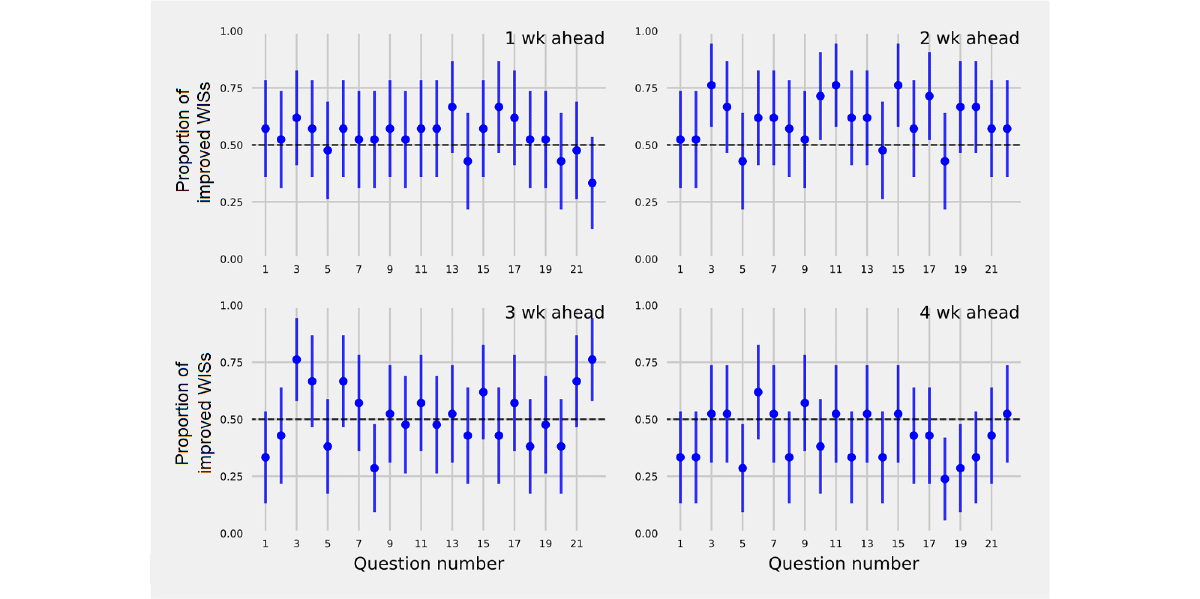
The proportion and 95% CIs of weighted interval scores (WISs) that were improved (smaller) for an SIR (susceptible, infected, and removed) plus vector autoregression moving average (VARMA) model that included mean perceived adherence (MEPA) time series 1-21 compared to the control SIR model without using a MEPA time series for forecasts 1-4 weeks ahead. An additional model, to the right of model number 21, is an SIR model plus a random forecast that includes all 21 MEPA time series and an ARIMA to model residuals. The majority of MEPA time series improved forecasts of incident cases 1 and 2 weeks ahead. A smaller number of MEPA time series improved forecasts 3 weeks ahead, and forecasts 4 weeks ahead were improved only modestly.

The proportion of times a forecast that included a single MEPA time series generated a smaller (improved) WIS compared to a model that did not use MEPA, was above 50% for the majority of adherence questions for forecast horizons of 1-3 weeks ahead (Figure 7). MEPA most improved forecasts 2 weeks ahead. The MEPA time series corresponding to the questions “What percent of people usually stay home?” “How common do people follow recommendations to receive antibody testing?” and “How common do people in your community follow guidelines to limit large gatherings?” improved 76% (95% CI 58%-94%) of forecasts 2 weeks ahead. For 3 weeks ahead, the question “What percent of people usually stay home?” improved 76% (95% CI 58%-94%) of forecasts and the machine learning model that incorporated all adherence questions improved 76% (95% CI 58%-94%) of forecasts. Including MEPA data improved forecasts 4 weeks ahead minimally and for only a small set of questions.

**Figure 8:**
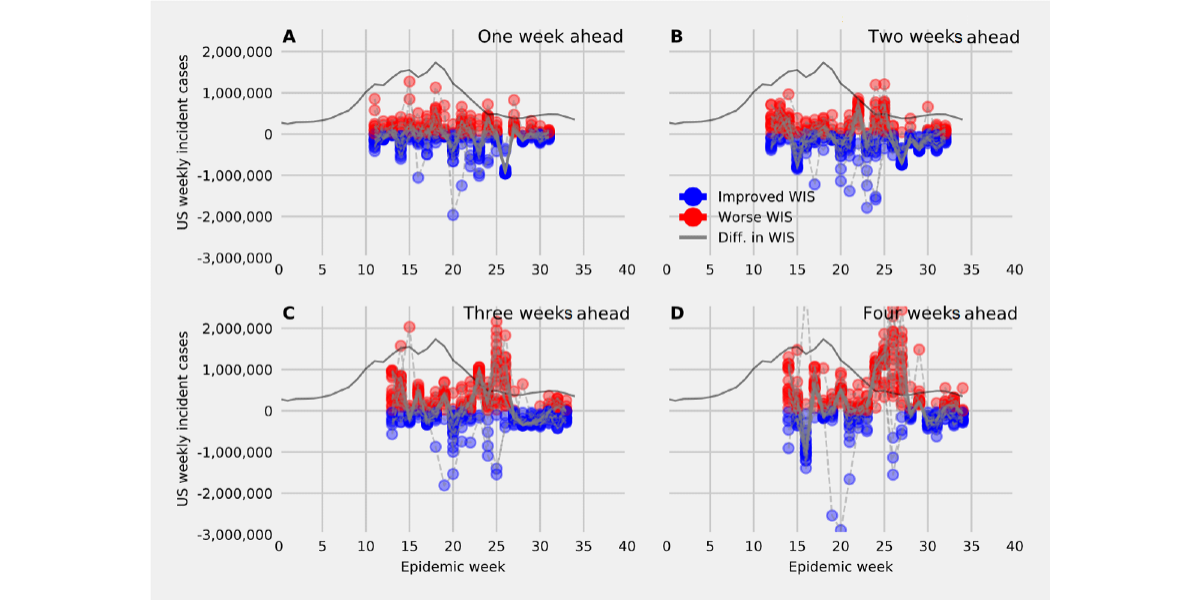
The differences in weighted interval scores (WISs) for forecasts of US national incident cases (A) 1 week ahead, (B) 2 weeks ahead, (C) 3 weeks ahead, and (D) 4 weeks ahead between models that included 1 mean perceived adherence time series and the control model that used only past incident case data to produce a forecast. The differences in WISs correspond to the forecasted epidemic weeks, not when the forecast was generated. The reported number of incident cases at the US national level is provided in grey. A point represents the difference in the WIS at the specific epidemic week and is colored red when a model weakens predictive performance and blue when this forecast improves upon the control model. Including perceptions of human behavior surrounding nonpharmaceutical interventions improves predictions at and after peak incident cases.

Compared with the control model, including MEPA data improved forecast accuracy 1-4 weeks ahead (ie, reduced WIS) at and after the peak reported number of incident cases (Figure 8). Forecasts 1 week ahead showed consistent small gains in forecast accuracy over time (Figure 8A). Forecasts 2 and 3 weeks ahead showed large gains in forecast accuracy at and just after the peak number of incident cases (Figure 8B and C), and improvements in forecast accuracy 4 weeks ahead appeared near the peak number of cases (Figure 8D). Overall, these results revealed that certain perceptions of NPI compliance can be useful signals in a model predicting COVID-19 incident cases.

**Figure 15:**
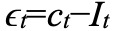
Inline graphic 7.

## Discussion

We found that crowdsourced perceptions of adherence to NPIs correlated with incident cases 1-4 weeks ahead at the US national level and that including perceptual data into a computational model improved forecast accuracy 1-3 weeks ahead. Because responses from a crowd can be collected quickly (ie, within hours of distributing an online survey), these responses can be included into a computational model that could provide real-time weekly forecasts of epidemiological targets to organizations such as the CDC.

Since forecasts based on public perceptions are rapid and informative, these forecasts would be highly effective at times following the issuing of new NPI guidelines from state or federal agencies to assess the effectiveness of these new guidelines. Our models could reveal the extent to which people perceive public compliance with these guidelines and how changes in compliance impact the trajectory of an infectious agent, thereby informing public health officials about which interventions are able to curtail risk-seeking behaviors. These forecasts may also be valuable for policy makers and community leaders as they decide, for example, whether college classes should be held in person or remotely.

This work supports the hypothesis that a crowd may be able to assign realistic probabilities to outcomes about community adherence to NPIs in line with recent work, which has shown that lay people can elicit accurate probabilistic predictions of diverse real-world phenomena such as box-office income of a new movie or the impact of an infectious agent [13,53]; however, much more work needs to be completed to assess to what degree including human judgment perceptions improves the predictive accuracy of an infectious disease model (Multimedia Appendix 7). Past literature about lay people’s ability to make accurate probabilistic predictions is mixed. Some past work suggests people may not be able to map environmental cues to accurate probabilities of outcomes [54], while other work has shown people’s statistical intuitions may overlap with the statistics of their environment [53].

Evidence from this study suggests that participants were able to gauge what activities they were able to observe and predict, and at what spatial level they could make predictions. For instance, participants were given the option to reply “Don’t know” or to leave questions blank. Participants responded more often to questions that were related to their environment, such as the proportion of people wearing masks, and responded less often to questions that were not related to their environment, such as restrictions on visitation to senior living centers. Survey questions during the initial pilot stage of the study asked participants to make predictions at the state level rather than community level, and many participants during this pilot stage protested that they could not make reasonable predictions at this level, suggesting that participants have some sense of how far a local community-level prediction could be extrapolated. Lastly, strong correlations between weekly responses to specific NPI questions indicated that the judgment of participants in this study was consistent (see Multimedia Appendix 5). Our results may support the idea that human judgment is predictive of incident cases because people can accurately perceive and make inferences about their surroundings.

However, relying on human judgment presents challenges that are absent when using computational models for prediction. Human judgment is susceptible to a wide array of biases often triggered by subtle changes in how a judgment prompt is presented [55]. Seemingly irrelevant information can have large impacts on judgment. For example, when asked to complete an irrelevant task, such as writing down the last 2 digits of their social security number before bidding on common items (such as a bottle of wine), people with higher social security numbers bid more money on wine than those with lower numbers [56]. Such findings underscore the importance of carefully crafting judgment questions to avoid activating judgment biases. Human judgment data must also be inspected for quality, as participants in this study often left one or more questions blank in a single survey and approximately 2% of participants gave the same response for every question, suggesting that they were not reading the survey items closely. Lastly, recruiting human participants demands time, effort, and money. Recruiting volunteers saves money but demands effort and implies an uncertain number of responses, which can be challenging when collecting data in response to a time-sensitive event such as an epidemic or pandemic. Participation rates in this study tended to increase throughout the data collection period, which created difficulties in assessing whether changes in MEPA over time were driven more by changes in perceived adherence or by changes in participation rates.

There are several limitations to address in future work. One limitation that we wish to overcome is that participants were not traced longitudinally, and so, we could not analyze how responses from individuals changed over time. Another limitation is that emails used to solicit volunteer participants contained a link to a summary of the findings from previous months of data collection. While this may have added value to a participant’s experience in the study, it may have biased their subsequent responses by anchoring their judgments to those summary values [56]. Another limitation arose from switching survey platforms (from SurveyMonkey to Pollfish) in the midst of data collection. The need for this switch was driven by a sudden decrease in the ability of SurveyMonkey to provide the requested number of paid responses each week (see Figure 1A). This switch seemed to have an impact on the geographical representativeness of the sample, as Pollfish provided a less representative sample than SurveyMonkey. Because switching survey platforms was confounded with both number of responses and epidemic week, the impact that switching survey platforms may have had on responses is largely unclear. Additionally, variable sampling rates across states created difficulties in estimating predictions at the state level. Oversampling from states with lower populations would ensure that a predictive model has sufficient data for estimating reliable predictions. No other demographic information was consistently collected throughout the surveys, and so, we were not able to assess whether the sample was representative for other demographic dimensions. Finally, there is evidence to suggest that self-expression may vary by geographic location [57]. Future research should consider how location and surrounding demographics may impact perceptions by, for example, leading to an overestimation of the prevalence of mask wearing in more densely populated areas.

Future research should explore whether more accurate and calibrated predictions of incident cases from human judgments can be made by matching the spatial scale of the questions posed to the crowd with the epidemiological target of interest. Instead of predicting incident cases at the national level, much stronger connections may be observed between state- or community-level judgments and state- or community-level incident cases. For example, one could investigate whether the accuracy of forecasts depends on factors such as the geographical size of the state (eg, Texas vs Delaware) or ethnic diversity (eg, California vs West Virginia). Additionally, respondents could be asked to judge compliance specifically at the level of their county, and then, these judgments could be added to a model that produces county-level predictions. Strong predictions at this local level would be valuable for community leaders when deciding, for example, whether a town hall meeting should be in person or remote. A significant challenge to estimating these local predictions is collecting enough responses from a given community over time, which, as mentioned above, can be remedied by targeting and oversampling from areas of interest to make local predictions. Future research should also explore whether perceptions of NPI compliance can predict other epidemiological targets. While we focused on incident cases in this study, our current methods should scale to other prediction outcomes of interest, such as COVID-19 hospitalizations and deaths.

Crowdsourced perceptions of human behavior, such as nonpharmaceutical adherence, may be a fast and informative signal that can improve probabilistic forecasts of the trajectory of an infectious agent and may have important implications for policy around infectious diseases.

## Appendix

Multimedia Appendix 1 Survey provided to participants to capture perceptions of adherence to nonpharmaceutical interventions.

Multimedia Appendix 2 Observed and expected proportions of participants for each state.

Multimedia Appendix 3 Correlation between mean perceived adherence and US national incident cases.

Multimedia Appendix 4 SIR (susceptible, infected, and removed) model fit to US national incident cases.

Multimedia Appendix 5 Autocorrelation of 1 week for mean perceived adherence time series.

Multimedia Appendix 6 Bivariate relationships between question clusters and incident cases.

Multimedia Appendix 7 Hypothesis testing across models in forecasting with crowdsourced mean perceived adherence.

## Abbreviations

AIC: Akaike information criterion
ARIMA: autoregressive integrated moving average
CDC: Centers for Disease Control and Prevention
IRB: institutional review board
MEPA: mean perceived adherence
NPI: nonpharmaceutical intervention
SIR: susceptible, infected, and removed
VARMA: vector autoregression moving average
WIS: weighted interval score
