# Supplementary material for: Crowdsourced Perceptions of Human Behavior to Improve Computational Forecasts of US National Incident Cases of COVID-19: Survey Study
Source: JMIR Public Health Surveill. 2022 Dec 30;8(12):e39336. doi: 10.2196/39336 (PMC9822568; doi:10.2196/39336)

**Multimedia Appendix 1.** Survey provided to participants to capture perceptions of adherence to nonpharmaceutical interventions.


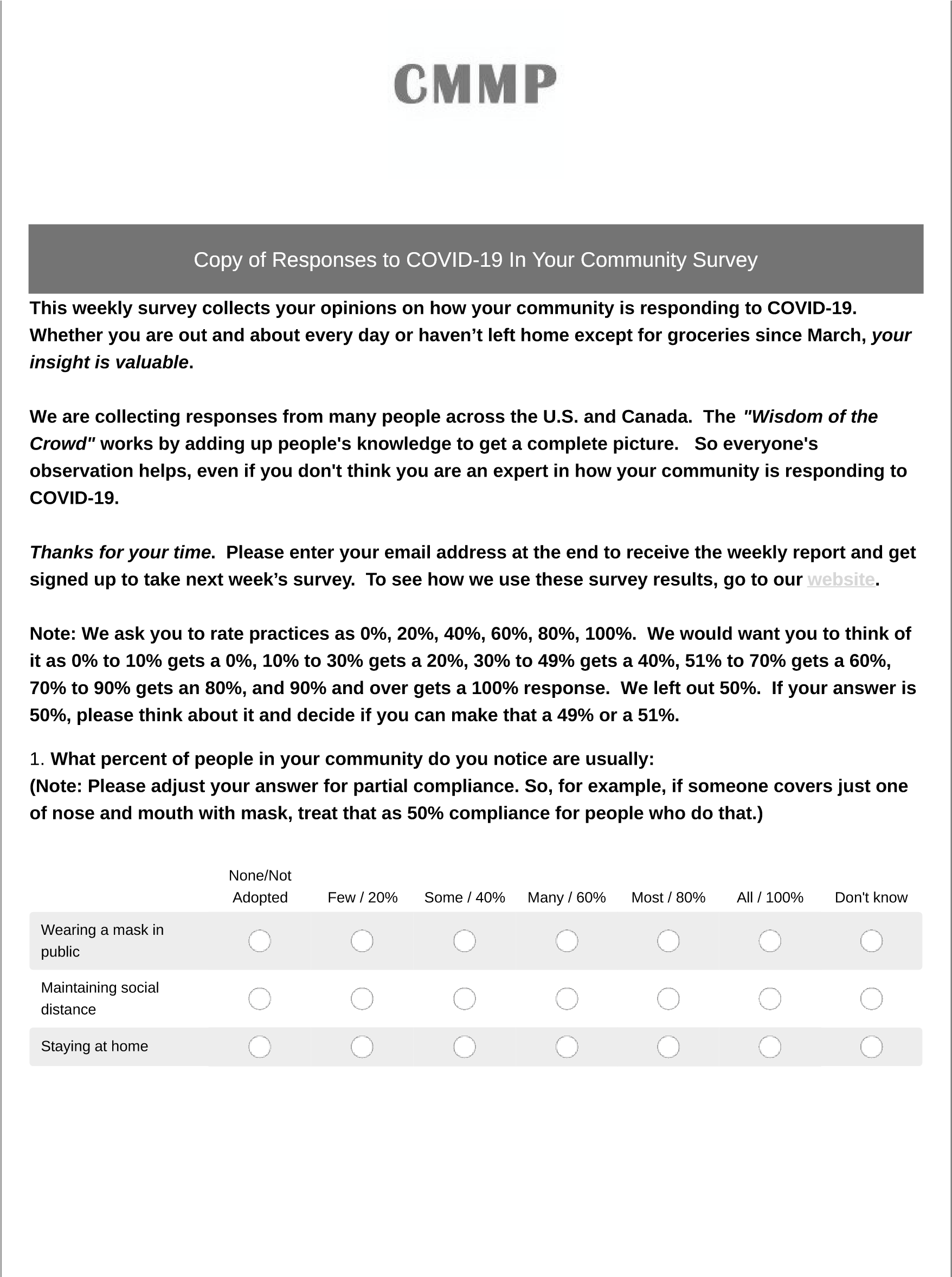


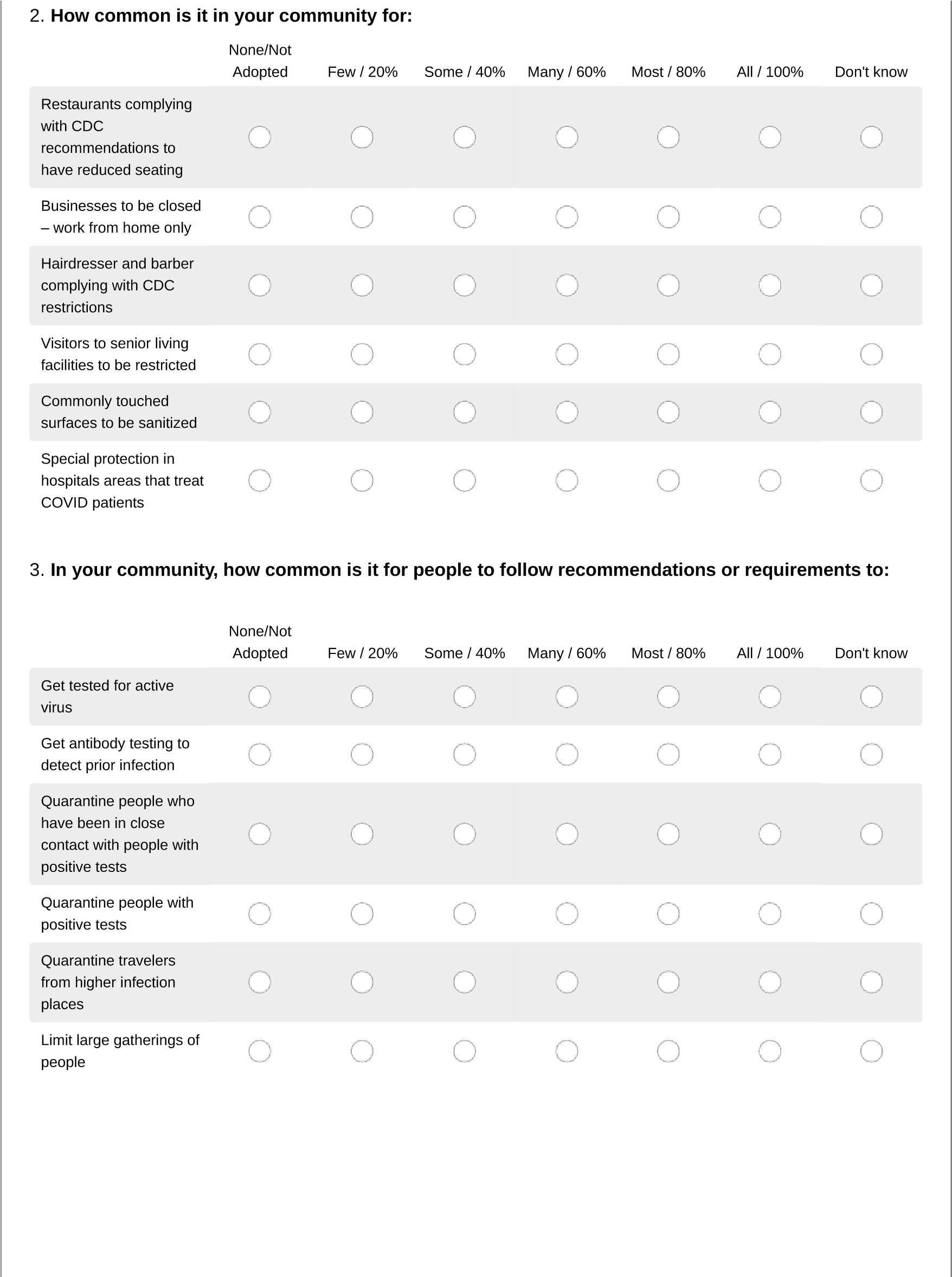


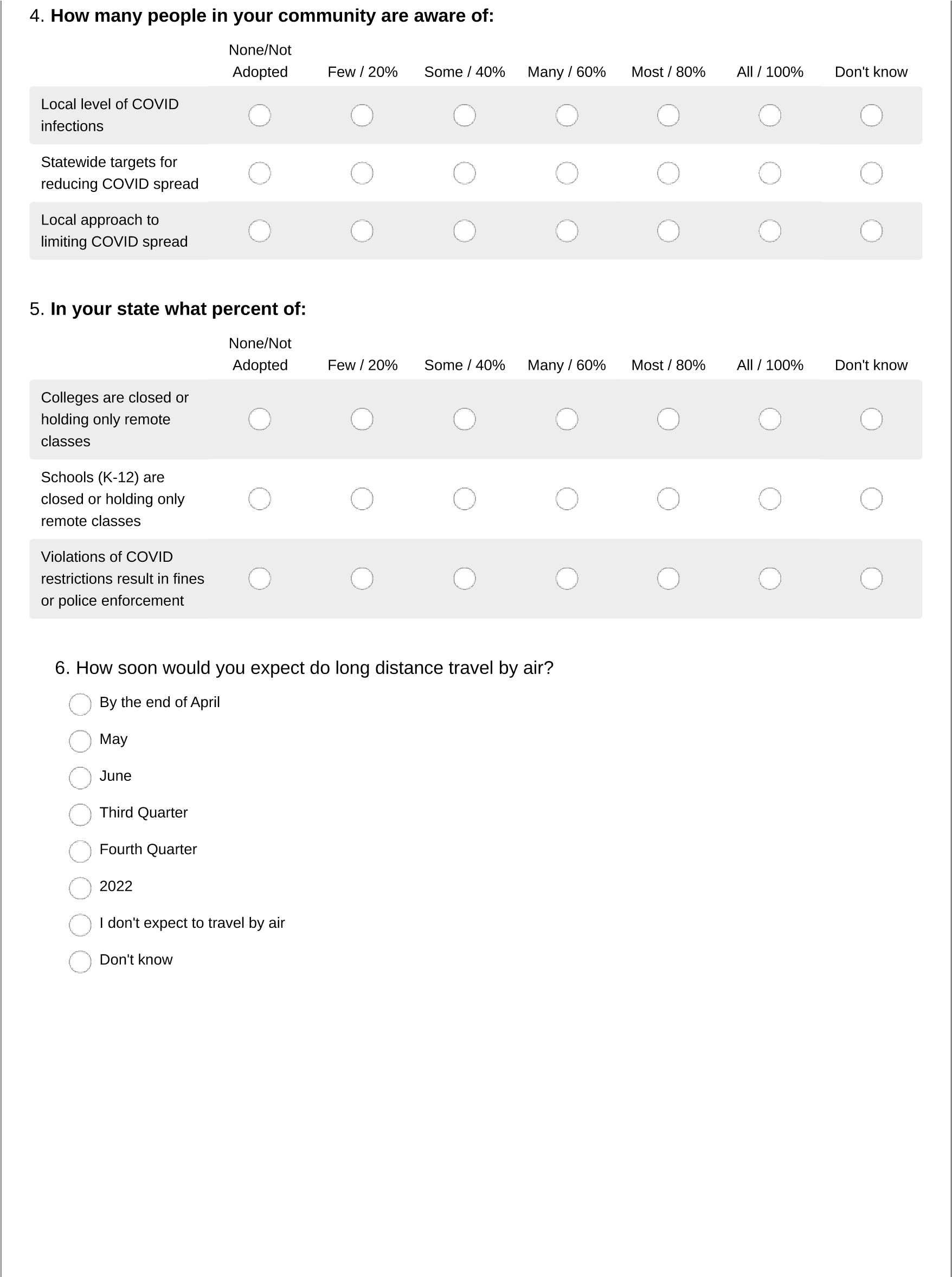


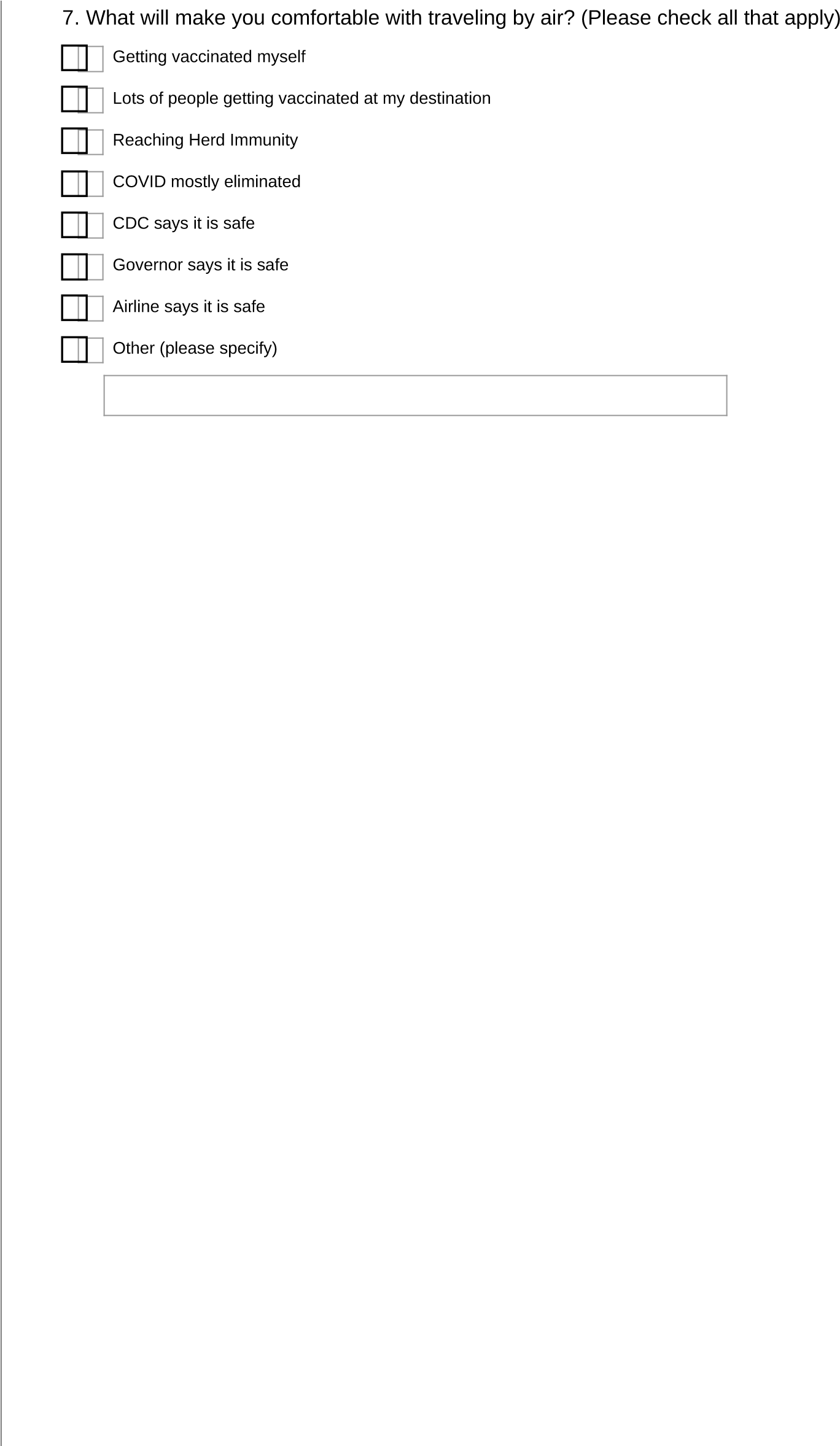


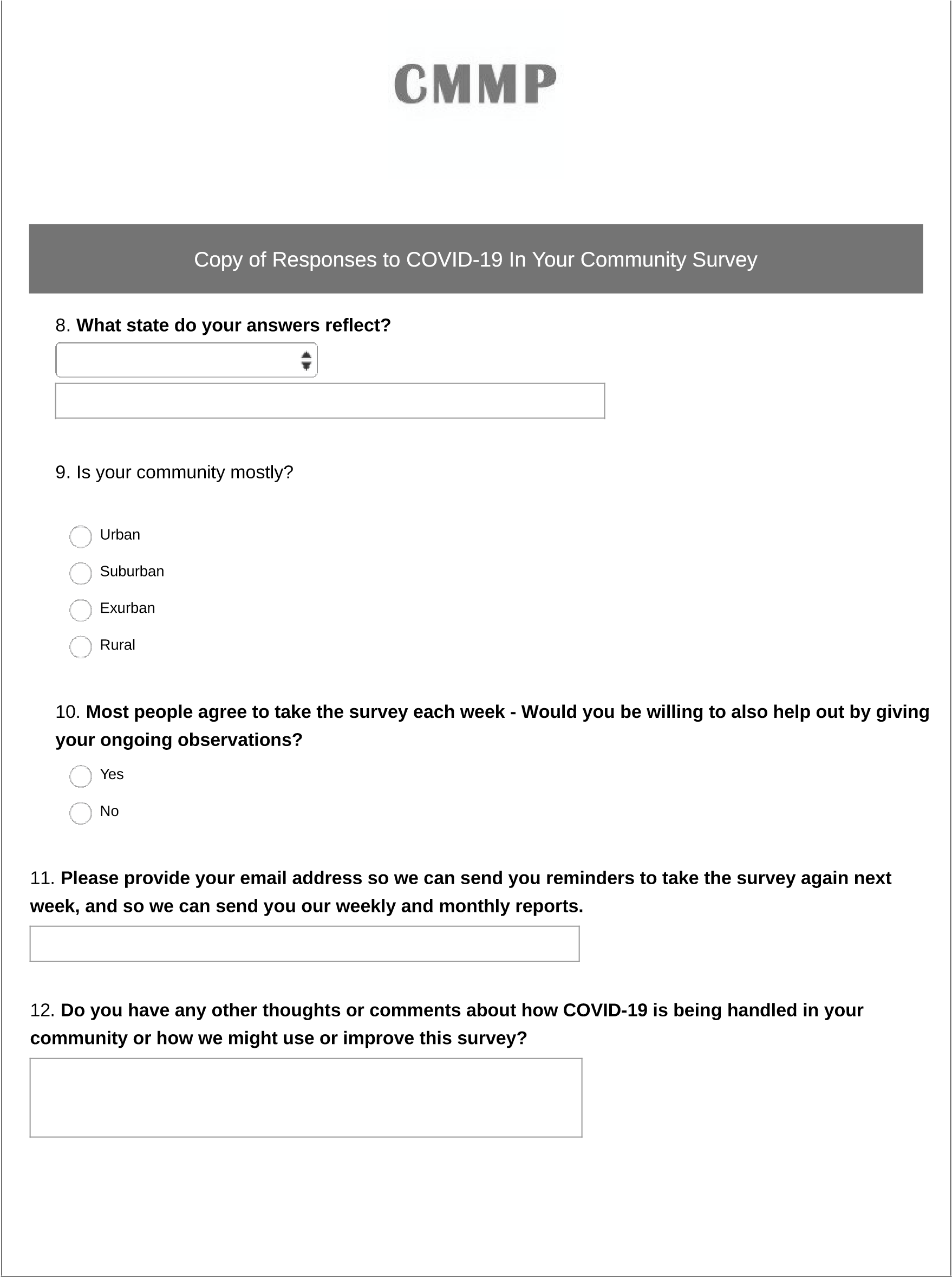

Supplement: Multimedia Appendix 1 [file publichealth_v8i12e39336_app1.docx]
