# Supplementary material for: Crowdsourced Perceptions of Human Behavior to Improve Computational Forecasts of US National Incident Cases of COVID-19: Survey Study
Source: JMIR Public Health Surveill. 2022 Dec 30;8(12):e39336. doi: 10.2196/39336 (PMC9822568; doi:10.2196/39336)
Supplement: Multimedia Appendix 2 [file publichealth_v8i12e39336_app2.docx]

**Multimedia Appendix 2.** Observed and expected proportions of participants for each state.

The observed and expected proportion according to the US national census, and relative difference between observed and expected proportions over the 36 week survey period stratified by state

| **State** | **Observed Proportion** | **Expected Proportion** | **Relative Difference** |
| --- | --- | --- | --- |
| Alabama | .0084 | .0149 | -0.44 |
| Alaska | .0016 | .0022 | -0.29 |
| Arizona | .0134 | .0222 | -0.40 |
| Arkansas | .0054 | .0092 | -0.42 |
| California | .1007 | .1204 | -0.16 |
| Colorado | .0124 | .0175 | -0.29 |
| Connecticut | .0223 | .0109 | 1.06 |
| Delaware | .0039 | .0030 | 0.31 |
| District Of Columbia | .0054 | .0022 | 1.50 |
| Florida | .0481 | .0654 | -0.27 |
| Georgia | .0320 | .0323 | -0.01 |
| Hawaii | .0015 | .0043 | -0.66 |
| Idaho | .0057 | .0054 | 0.05 |
| Illinois | .0360 | .0386 | -0.07 |
| Indiana | .0145 | .0205 | -0.29 |
| Iowa | .0121 | .0096 | 0.26 |
| Kansas | .0072 | .0089 | -0.19 |
| Kentucky | .0150 | .0136 | 0.10 |
| Louisiana | .0145 | .0142 | 0.03 |
| Maine | .0061 | .0041 | 0.49 |
| Maryland | .0145 | .0184 | -0.21 |
| Massachusetts | .0287 | .0210 | 0.36 |
| Michigan | .0272 | .0304 | -0.11 |
| Minnesota | .0374 | .0172 | 1.18 |
| Mississippi | .0033 | .0091 | -0.64 |
| Missouri | .0138 | .0187 | -0.26 |
| Montana | .0019 | .0033 | -0.42 |
| Nebraska | .0079 | .0059 | 0.34 |
| Nevada | .0068 | .0094 | -0.27 |
| New Hampshire | .0025 | .0041 | -0.39 |
| New Jersey | .0411 | .0271 | 0.52 |
| New Mexico | .0026 | .0064 | -0.59 |
| New York | .0923 | .0593 | 0.56 |
| North Carolina | .0240 | .0320 | -0.25 |
| North Dakota | .0013 | .0023 | -0.46 |
| Ohio | .0414 | .0356 | 0.16 |
| Oklahoma | .0073 | .0121 | -0.40 |
| Oregon | .0095 | .0128 | -0.26 |
| Pennsylvania | .0714 | .0390 | 0.83 |
| Puerto Rico | .0017 | .0097 | -0.83 |
| Rhode Island | .0044 | .0032 | 0.37 |
| South Carolina | .0091 | .0157 | -0.42 |
| South Dakota | .0009 | .0027 | -0.65 |
| Tennessee | .0183 | .0208 | -0.12 |
| Texas | .0680 | .0883 | -0.23 |
| Utah | .0071 | .0098 | -0.28 |
| Vermont | .0012 | .0019 | -0.39 |
| Virginia | .0204 | .0260 | -0.21 |
| Washington | .0336 | .0232 | 0.45 |
| West Virginia | .0030 | .0055 | -0.46 |
| Wisconsin | .0290 | .0177 | 0.63 |
| Wyoming | .0021 | .0018 | 0.20 |
