# Supplementary material for: Crowdsourced Perceptions of Human Behavior to Improve Computational Forecasts of US National Incident Cases of COVID-19: Survey Study
Source: JMIR Public Health Surveill. 2022 Dec 30;8(12):e39336. doi: 10.2196/39336 (PMC9822568; doi:10.2196/39336)
Supplement: Multimedia Appendix 3 [file publichealth_v8i12e39336_app3.docx]

**Multimedia Appendix 3.** Correlation between mean perceived adherence and US national incident cases.

Estimated correlation coefficients (ˆρ) and 95% confidence intervals between MEPA time series and one through four week ahead US national incident cases

| **Question**  Week 0 | ***ρ*ˆ** | **Lower CI Upper CI** | |
| --- | --- | --- | --- |
| 1 | -0.094 | -0.414 | 0.247 |
| 2 | -0.467 | -0.692 | -0.158 |
| 3 | 0.051 | -0.287 | 0.378 |
| 4 | -0.018 | -0.349 | 0.317 |
| 5 | -0.285 | -0.565 | 0.053 |
| 6 | -0.194 | -0.495 | 0.149 |
| 7 | 0.021 | -0.315 | 0.352 |
| 8 | -0.104 | -0.423 | 0.237 |
| 9 | -0.068 | -0.392 | 0.272 |
| 10 | 0.002 | -0.331 | 0.335 |
| 11 | -0.136 | -0.449 | 0.207 |
| 12 | -0.203 | -0.502 | 0.140 |
| 13 | -0.080 | -0.403 | 0.260 |
| 14 | -0.280 | -0.561 | 0.059 |
| 15 | -0.342 | -0.606 | -0.009 |
| 16 | -0.059 | -0.385 | 0.280 |
| 17 | -0.029 | -0.359 | 0.307 |
| 18 | -0.394 | -0.642 | -0.069 |
| 19 | 0.518 | 0.223 | 0.726 |
| 20 | 0.335 | 0.002 | 0.601 |
| 21 Week 1 | -0.249 | -0.538 | 0.092 |
| 1 | -0.099 | -0.418 | 0.242 |
| 2 | -0.459 | -0.687 | -0.148 |
| 3 | -0.089 | -0.410 | 0.252 |
| 4 | 0.084 | -0.257 | 0.406 |
| 5 | -0.348 | -0.610 | -0.017 |
| 6 | -0.100 | -0.419 | 0.241 |
| 7 | 0.046 | -0.292 | 0.373 |
| 8 | -0.071 | -0.395 | 0.269 |
| 9 | -0.003 | -0.336 | 0.330 |
| 10 | -0.082 | -0.404 | 0.258 |
| 11 | -0.201 | -0.501 | 0.142 |
| 12 | -0.125 | -0.440 | 0.218 |
| 13 | -0.011 | -0.343 | 0.324 |
| 14 | -0.328 | -0.596 | 0.006 |
| 15 | -0.296 | -0.573 | 0.041 |
| 16 | -0.101 | -0.420 | 0.241 |
| 17 | -0.094 | -0.415 | 0.247 |
| 18 | -0.403 | -0.649 | -0.081 |
| 19 | 0.458 | 0.147 | 0.686 |
| 20 | 0.273 | -0.066 | 0.556 |
| 21 | -0.305 | -0.579 | 0.031 |

| Week 2 | |  | |  |  | |
| --- | --- | --- | --- | --- | --- | --- |
| 1 | | -0.144 | | -0.455 | 0.199 | |
| 2 | | -0.433 | | -0.670 | -0.117 | |
| 3 | | -0.237 | | -0.528 | 0.105 | |
| 4 | | 0.185 | | -0.158 | 0.488 | |
| 5 | | -0.393 | | -0.642 | -0.069 | |
| 6 | | -0.025 | | -0.355 | 0.311 | |
| 7 | | 0.089 | | -0.252 | 0.410 | |
| 8 | | 0.005 | | -0.329 | 0.338 | |
| 9 | | 0.071 | | -0.268 | 0.395 | |
| 10 | | -0.185 | | -0.488 | 0.158 | |
| 11 | | -0.275 | | -0.557 | 0.064 | |
| 12 | | -0.124 | | -0.439 | 0.219 | |
| 13 | | 0.033 | | -0.303 | 0.363 | |
| 14 | | -0.400 | | -0.647 | -0.077 | |
| 15 | | -0.245 | | -0.534 | 0.096 | |
| 16 | | -0.148 | | -0.459 | 0.195 | |
| 17 | | -0.182 | | -0.486 | 0.161 | |
| 18 | | -0.390 | | -0.640 | -0.065 | |
| 19 | | 0.364 | | 0.035 | 0.622 | |
| 20 | | 0.225 | | -0.117 | 0.519 | |
| 21 Week 3 | | -0.356 | | -0.616 | -0.026 | |
| 1 | | -0.116 | | -0.433 | 0.226 | |
| 2 | | -0.347 | | -0.610 | -0.016 | |
| 3 | | -0.341 | | -0.605 | -0.008 | |
| 4 | | 0.310 | | -0.026 | 0.583 | |
| 5 | | -0.355 | | -0.615 | -0.024 | |
| 6 | | 0.091 | | -0.250 | 0.412 | |
| 7 | | 0.160 | | -0.183 | 0.468 | |
| 8 | | 0.096 | | -0.245 | 0.416 | |
| 9 | | 0.181 | | -0.162 | 0.485 | |
| 10 | | -0.252 | | -0.540 | 0.089 | |
| 11 | | -0.365 | | -0.622 | -0.036 | |
| 12 | | -0.081 | | -0.403 | 0.259 | |
| 13 | | 0.128 | | -0.214 | 0.442 | |
| 14 | | -0.460 | | -0.688 | -0.150 | |
| 15 | | -0.127 | | -0.442 | 0.215 | |
| 16 | | -0.198 | | -0.498 | 0.145 | |
| 17 | | -0.270 | | -0.553 | 0.070 | |
| 18 | | -0.352 | | -0.613 | -0.021 | |
| 19 | | 0.268 | | -0.072 | 0.552 | |
| 20 | | 0.157 | | -0.186 | 0.466 | |
| 21 Week 4 | | -0.418 | | -0.659 | -0.098 | |
| 1 | | -0.085 | | -0.407 | 0.256 | |
| 2 | | -0.259 | | -0.545 | 0.081 | |
| 3 | | -0.434 | | -0.670 | -0.118 | |
| 4 | | 0.440 | | 0.125 | 0.674 | |
| 5 -0.357 | | -0.617 | | | -0.027 | |
| 6 0.237 | | -0.105 | | | 0.528 | |
| 7 0.264 | | -0.075 | | | 0.549 | |
| 8 0.148 | | -0.194 | | | 0.459 | |
| 9 0.300 | | -0.037 | | | 0.576 | |
| 10 -0.320 | | -0.591 | | | 0.014 | |
| 11 -0.447 | | -0.679 | | | -0.134 | |
| 12 0.068 | | -0.272 | | | 0.392 | |
| 13 0.258 | | -0.083 | | | 0.544 | |
| 14 -0.509 | | -0.720 | | | -0.211 | |
| 15 0.040 | | -0.297 | | | 0.368 | |
| 16 -0.279 | | -0.560 | | | 0.060 | |
| 17 -0.358 | | -0.618 | | | -0.028 | |
| 18 -0.289 | | -0.568 | | | 0.049 | |
| 19 0.151 | | -0.192 | | | 0.461 | |
| 20 0.023 | | -0.313 | | | 0.354 | |
| 21 -0.507 | | -0.719 | | | -0.209 | |
