# Supplementary material for: Crowdsourced Perceptions of Human Behavior to Improve Computational Forecasts of US National Incident Cases of COVID-19: Survey Study
Source: JMIR Public Health Surveill. 2022 Dec 30;8(12):e39336. doi: 10.2196/39336 (PMC9822568; doi:10.2196/39336)
Supplement: Multimedia Appendix 4 [file publichealth_v8i12e39336_app4.docx]

**Multimedia Appendix 4.** SIR (susceptible, infected, and removed) model fit to US national incident cases.

The number of US national incident cases that are observed during a specific week (black), future unobserved incident cases (gray), and a simple SIR model (blue) fitted at survey week (A.) 10 which corresponds to epidemic 202036, survey week (B.) 21, (C.) 25, and (D.) 35 out of the 36 weeks that this survey was conducted.

2020-09

2020-11

2021-01

2021-04

500,000

1,000,000

1,500,000

2,000,000

2,500,000

Incident cases US

**A.**

Survey week = 10/36

2020-09

2020-11

2021-01

2021-04

500,000

1,000,000

1,500,000

2,000,000

2,500,000

Incident cases US

**B.**

Survey week = 21/36

2020-09

2020-11

2021-01

2021-04

500,000

1,000,000

1,500,000

2,000,000

2,500,000

Incident cases US

**C.**

Survey week = 25/36

2020-09

2020-11

2021-01

2021-04

500,000

1,000,000

1,500,000

2,000,000

2,500,000

Incident cases US

**D.**

Survey week = 35/36
