# Supplementary material for: Crowdsourced Perceptions of Human Behavior to Improve Computational Forecasts of US National Incident Cases of COVID-19: Survey Study
Source: JMIR Public Health Surveill. 2022 Dec 30;8(12):e39336. doi: 10.2196/39336 (PMC9822568; doi:10.2196/39336)

**Multimedia Appendix 5.** Autocorrelation of 1 week for mean perceived adherence time series.

Auto correlation between MEPA at week *w* + 1 and MEPA at week *w* for all twenty one questions over the survey period. The correlation is high for the majority of MEPA time series, suggesting MEPA could be modeled by an auto regressive process and contains more structure than a random walk.


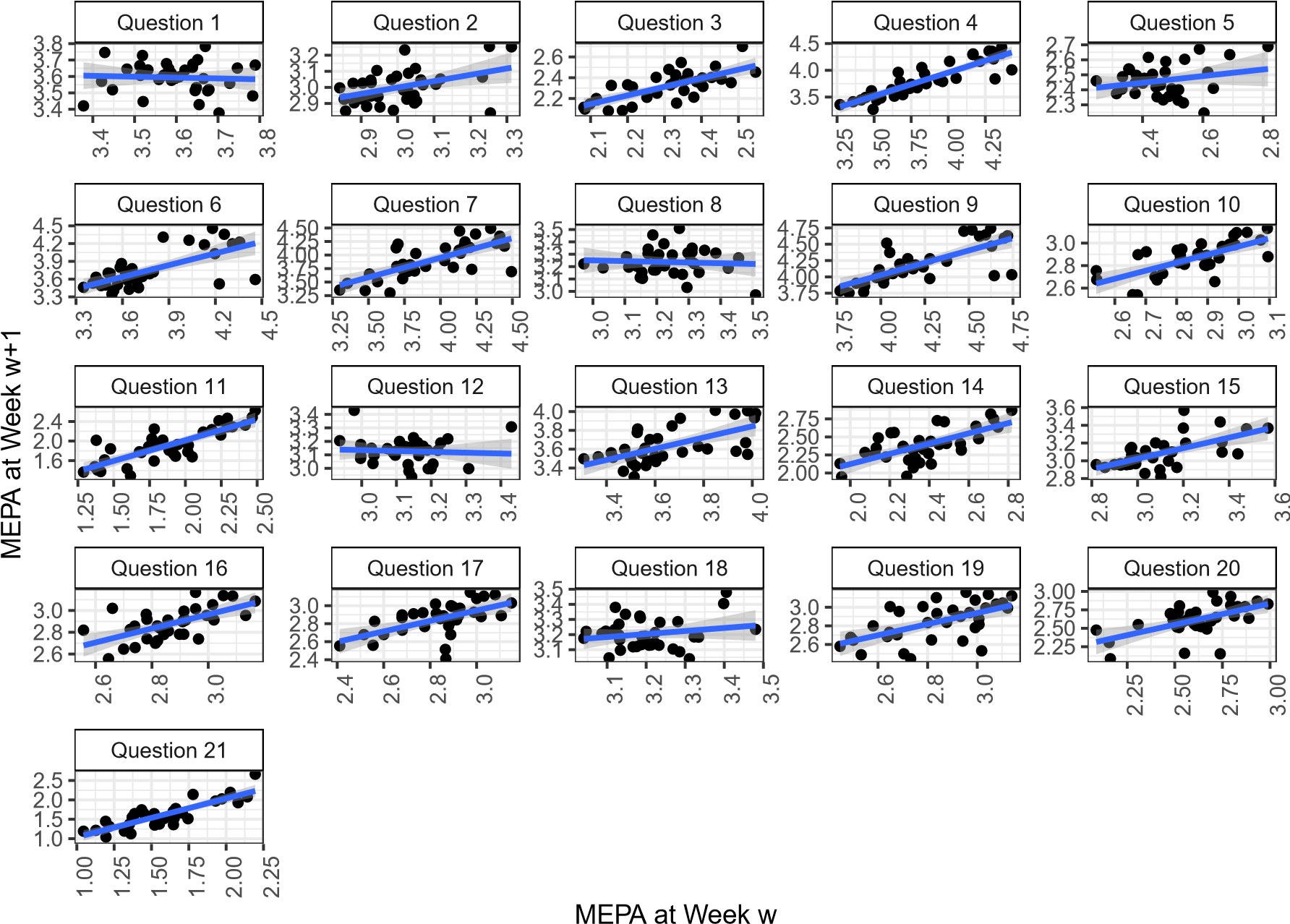

Supplement: Multimedia Appendix 5 [file publichealth_v8i12e39336_app5.docx]
