# Supplementary material for: Crowdsourced Perceptions of Human Behavior to Improve Computational Forecasts of US National Incident Cases of COVID-19: Survey Study
Source: JMIR Public Health Surveill. 2022 Dec 30;8(12):e39336. doi: 10.2196/39336 (PMC9822568; doi:10.2196/39336)

**Multimedia Appendix 6.** Bivariate relationships between question clusters and incident cases.

Bivariate relationships between four clusters of MEPA time series and one through four week ahead incident cases at the US national level.


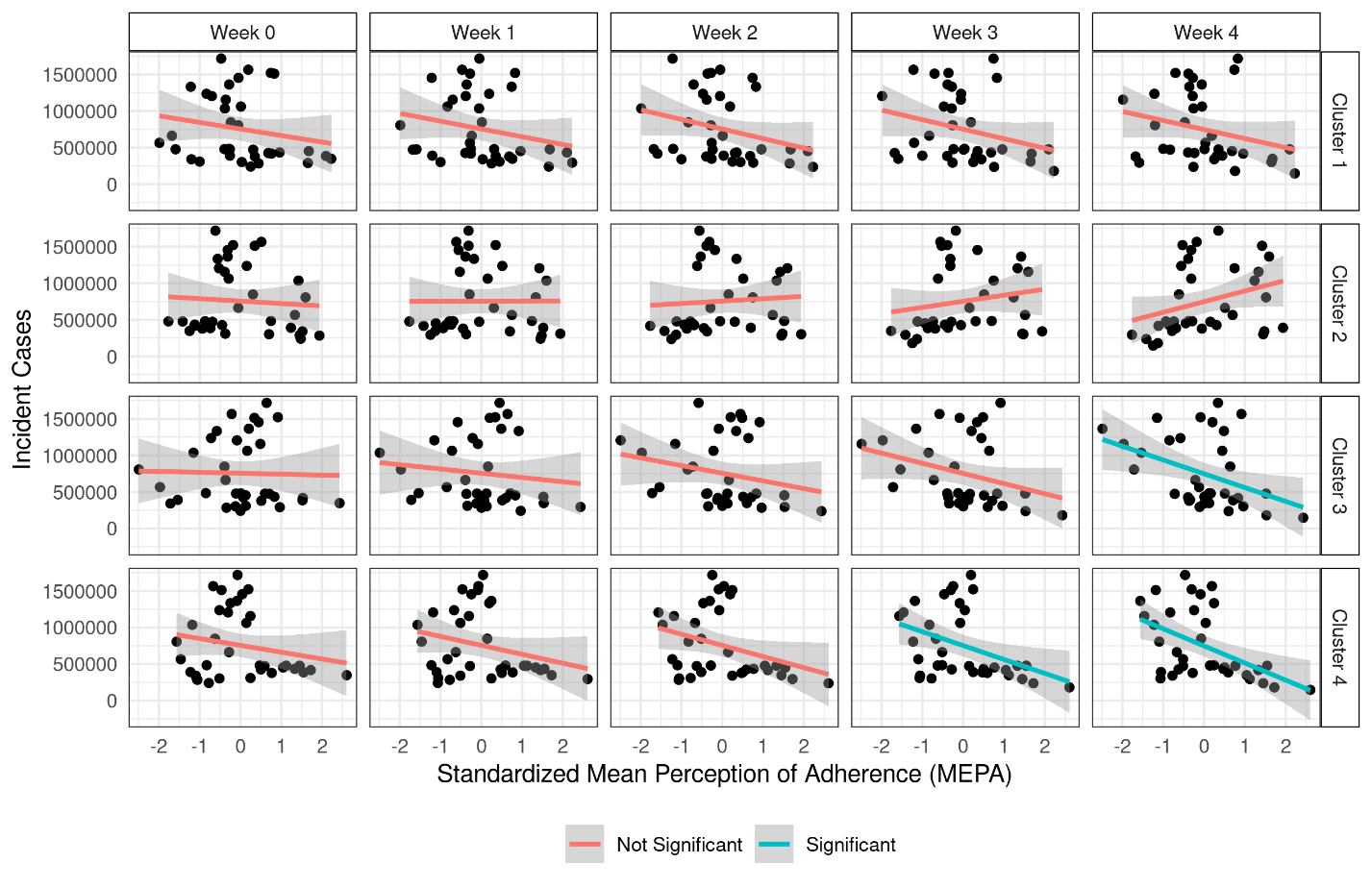

Supplement: Multimedia Appendix 6 [file publichealth_v8i12e39336_app6.docx]
