# Supplementary material for: Crowdsourced Perceptions of Human Behavior to Improve Computational Forecasts of US National Incident Cases of COVID-19: Survey Study
Source: JMIR Public Health Surveill. 2022 Dec 30;8(12):e39336. doi: 10.2196/39336 (PMC9822568; doi:10.2196/39336)
Supplement: Multimedia Appendix 7 [file publichealth_v8i12e39336_app7.docx]

**Multimedia Appendix 7.** Hypothesis testing across models in forecasting with crowdsourced mean perceived adherence.

We compared the WIS score between models that included human perception data vs a reference model that included only reported surveillance data for all twenty-one questions and for all four time horizons (1 week ahead, 2 weeks ahead, etc). Each pair of WIS scores, one for the model including MEPA and one without, can be mapped to the value one if the model with MEPA produced a smaller (improved) WIS score compared to the model without MEPA and to value zero if this model produced a larger (worse) value.

We expect the observed ones and zeros to follow a Binomial distribution with parameter p and can assess the following hypothesis

$$H_{null}=p<\frac{1}{2}$$

$$H_{alternative}=p\geq\frac{1}{2}$$

with an exact Binomial test. Below we report the pvalue for all questions and time horizons. A pvalue less than 0.10 indicates that including this MEPA time series may improve predictions of the number of incident cases of COVID-19 at the US national level.

|  | One Week Ahead | Two Weeks Ahead | Three Weeks Ahead | Four Weeks Ahead |
| --- | --- | --- | --- | --- |
| 1 | 0.33 | 0.50 | 0.96 | 0.96 |
| 2 | 0.50 | 0.5 | 0.81 | 0.96 |
| 3 | 0.19 | 0.01 | 0.01 | 0.50 |
| 4 | 0.33 | 0.09 | 0.09 | 0.50 |
| 5 | 0.67 | 0.81 | 0.91 | 0.99 |
| 6 | 0.33 | 0.19 | 0.09 | 0.19 |
| 7 | 0.50 | 0.19 | 0.33 | 0.50 |
| 8 | 0.50 | 0.33 | 0.99 | 0.96 |
| 9 | 0.33 | 0.50 | 0.50 | 0.33 |
| 10 | 0.50 | 0.04 | 0.67 | 0.91 |
| 11 | 0.33 | 0.01 | 0.33 | 0.50 |
| 12 | 0.33 | 0.19 | 0.67 | 0.96 |
| 13 | 0.09 | 0.19 | 0.50 | 0.50 |
| 14 | 0.81 | 0.67 | 0.81 | 0.96 |
| 15 | 0.33 | 0.01 | 0.19 | 0.50 |
| 16 | 0.09 | 0.33 | 0.81 | 0.81 |
| 17 | 0.19 | 0.04 | 0.33 | 0.81 |
| 18 | 0.50 | 0.81 | 0.91 | 1.00 |
| 19 | 0.50 | 0.09 | 0.67 | 0.99 |
| 20 | 0.81 | 0.09 | 0.91 | 0.96 |
| 21 | 0.67 | 0.33 | 0.09 | 0.81 |
| 22 | 0.96 | 0.33 | 0.01 | 0.50 |
